# Anti-LGI1 encephalitis: clinical presentation, imaging features, and prognostic analysis

**DOI:** 10.3389/fneur.2025.1703647

**Published:** 2025-12-15

**Authors:** Xin Chen, Qiuyan Liu, Xiaojuan Wang, Songke Lu, Shaomin Zuo, Han Zheng, Hongzhi Guan, Wei Li

**Affiliations:** 1 Henan Medical University, Henan Provincial People’s Hospital, Zhengzhou, China; 2 Department of Neurology, Henan Provincial People's Hospital, Zhengzhou, China; 3 Department of Neurology, People’s Hospital of Zhengzhou University, Zhengzhou, China; 4 Department of Neurology, People's Hospital of Henan University, Zhengzhou, China; 5 Department of Neurology, Peking Union Medical College Hospital, Chinese Academy of Medical Sciences, Beijing, China

**Keywords:** autoimmune encephalitis, anti-leucine-rich glioma-inactivated 1 antibody, encephalitis, immunotherapy, prognosis

## Abstract

**Objective:**

To investigate the clinical features, neuroimaging characteristics, and prognosis of patients with anti–leucine-rich glioma-inactivated 1 (LGI1) antibody associated encephalitis.

**Methods:**

We conducted a retrospective study of 87 patients diagnosed with anti-LGI1 encephalitis during the acute phase, admitted to two tertiary hospitals in China between January 2022 and September 2024. Clinical data, neuroimaging findings, and follow-up outcomes were systematically analyzed.

**Results:**

The primary clinical manifestations included memory impairment, epileptic seizures, psychiatric and behavioral disturbances, sleep dysfunction, involuntary movements, faciobrachial dystonic seizures (FBDS), and autonomic dysfunction. Among 53 patients tested for thyroid function, 9 (17.0%) exhibited abnormalities. Hyponatremia was observed in 41 of 82 patients (50.0%), and hyperhomocysteinemia (HHCY) in 23 of 80 (28.7%). Among 68 patients who underwent cerebrospinal fluid (CSF) analysis, elevated intracranial pressure was observed in 10 (14.7%), pleocytosis in 22 (32.4%), and elevated protein levels in 27 (39.7%). Electrocardiographic abnormalities were identified in 26 of 42 patients (61.9%) without prior cardiovascular disease. MRI abnormalities were present in 75 of 80 patients (93.8%), most commonly affecting the temporal lobe (55.0%), hippocampus (51.2%), and amygdala (11.2%). PET imaging in 39 patients frequently revealed hypermetabolism in the basal ganglia and temporal lobe. All patients received first-line immunotherapy. Of the 63 patients with follow-up data, 59 (93.7%) achieved favorable outcomes.

**Conclusion:**

Anti-LGI1 encephalitis is an immune-mediated autoimmune disorder characterized by memory impairment, epileptic seizures, FBDS, psychiatric and behavioral disturbances, autonomic dysfunction, hyponatremia, and HHCY. Immunotherapy is generally effective, leading to favorable prognosis in the majority of patients.

## Introduction

Autoimmune encephalitis (AE) comprises a group of inflammatory neurological disorders mediated by autoantibodies, characterized by focal or widespread involvement of the central nervous system. Epidemiological studies indicate that anti–N-methyl-D-aspartate receptor (anti-NMDAR) encephalitis is the most prevalent subtype, accounting for approximately 54–80% of AE cases (1). Encephalitis associated with antibodies against leucine-rich glioma-inactivated 1 (LGI1) protein, often classified as limbic encephalitis due to its predominant involvement of the limbic system (2), is relatively rare, with an estimated annual incidence of 0.83 per 1 million individuals (3), and typically exhibits an insidious onset. Notably, different antibody-mediated subtypes of AE present with distinct clinical manifestations.

In recent years, advances in antibody detection techniques have facilitated the accurate diagnosis of an increasing number of anti-LGI1 encephalitis cases. Nevertheless, the clinical features, laboratory findings, and neuroimaging characteristics in these patients may differ from those reported in earlier studies. Currently, the diagnosis of AE relies heavily on antibody testing. Although antibody detection is convenient for clinicians, it often requires considerable time. Therefore, in addition to the identification of characteristic antibodies, careful evaluation of clinical manifestations remains equally critical for accurate diagnosis.

Therefore, this study retrospectively analyzed the clinical data of patients with anti-LGI1 encephalitis who were admitted to the neurology departments of two tertiary hospitals in northern China. The primary objective was to systematically characterize the clinical features, laboratory parameters, and neuroimaging findings of these patients. Additionally, we aim to identify key features of the disease, enhance clinicians’ understanding of anti-LGI1 encephalitis, and provide valuable evidence for accurate diagnosis and the development of therapeutic strategies. Furthermore, this study seeks to improve early recognition and optimize personalized management strategies for this immune-mediated encephalitis.

## Materials and methods

### Study subjects

Clinical data were retrospectively collected from 87 patients diagnosed with anti-LGI1 encephalitis and admitted to the Department of Neurology at Peking Union Medical College Hospital and the People’s Hospital of Zhengzhou University between January 2022 and September 2024. Patients were diagnosed with anti-LGI1 encephalitis according to the Chinese expert consensus on the diagnosis and management of autoimmune encephalitis (2022 edition) (4). The inclusion and exclusion criteria were as follows: (1) acute or subacute onset with one or more of the following clinical manifestations—working memory impairment, seizures, or psychiatric and behavioral abnormalities, etc.; (2) positivity for LGI1-IgG antibodies in serum and/or CSF, confirmed by both cell-based assay (CBA) and tissue-based assay (TBA); (3) exclusion of patients with other AE-related antibodies, infectious encephalopathies, primary psychiatric disorders, or metabolic encephalopathies.

### Study methods

#### Clinical data collection

Basic demographic and clinical information was collected, including age at onset, sex, clinical manifestations, CSF parameters, thyroid function, brain MRI and whole-body PET/CT findings, treatment strategies, and clinical outcomes. In accordance with previous studies (5), neurological function was assessed using the modified Rankin Scale (mRS) at hospital admission and at the last follow-up. A favorable prognosis was defined as an mRS score ≤ 2, whereas an unfavorable prognosis was defined as an mRS score > 2.

##### Neuroimaging

Cranial MRI was performed using 3.0 T scanners (Siemens), including axial T1-weighted, T2-weighted, fluid-attenuated inversion recovery (FLAIR), diffusion weighted imaging(DWI), and apparent diffusion coefficient imaging(ADC) sequences. PET/CT refers specifically to 18F-fluoridesoxyglucose-positron emission tomography (18F-FDG-PET/CT, Siemens Biograph mCT).

Thyroid function tests included measurements of triiodothyronine (T3), thyroxine (T4), free triiodothyronine (FT3), free thyroxine (FT4), thyroid-stimulating hormone (TSH), thyroglobulin antibody (TGAb), and anti-thyroid peroxidase antibody (TPOAb). In this study, abnormal thyroid function was defined as any of these parameters exceeding or falling below their respective reference ranges.

#### Treatment strategy

(1) Acute-phase treatment: First-line immunotherapy included high-dose corticosteroids [500–1,000 mg/day for adults; 30 mg/(kg·day) for children for 3–5 days, followed by gradual tapering], intravenous immunoglobulin (IVIG) [0.4 g/(kg·day) for 5 consecutive days], or plasma exchange. Second-line immunotherapy consisted of rituximab or cyclophosphamide.
(2) Maintenance therapy: Maintenance treatment included corticosteroid tapering and oral administration of mycophenolate mofetil.

#### Definition of relapse

Relapse was defined as the recurrence of new neurological symptoms or the worsening of pre-existing symptoms after at least 2 months of clinical stability or improvement, or as radiological evidence of disease progression.

#### Antibody testing

The presence of neuronal autoantibodies in both serum and CSF was assessed using CBA and TBA. In the CBA, transfected cells expressing neuronal cell-surface antigens served as the antigen substrate, whereas in the TBA, animal brain tissue sections were utilized. All antibody testing was performed at the V-MEDICAL Laboratory.

The panel of antibodies tested included anti-NMDAR, anti-LGI1, anti-GABAAR, anti-GABABR, anti-CASPR2, anti-IgLON5, anti-AMPAR, anti-DPPX, anti-mGluR5, anti-D2R, anti-GlyR, and anti-GAD antibodies. Additionally, onconeural antibodies in serum and CSF, including anti-Hu, anti-Ri, anti-Yo, anti-CV2/CRMP5, anti-Ma2, and anti-amphiphysin antibodies, were also evaluated.

### Statistical analysis

Normality of continuous variables was assessed prior to analysis. Data with a normal distribution are presented as mean ± standard deviation (x̄ ± s) and were compared using the independent-samples *t*-test. Non-normally distributed data are presented as median and interquartile range [M (Q1, Q3)] and were compared using the Mann–Whitney U test. For paired data that followed a normal distribution, differences between pre-treatment and post-treatment scores were analyzed using the paired *t*-test. For non-normally distributed data, the Wilcoxon signed-rank test was applied. Categorical variables are reported as frequencies and percentages [*n* (%)] and were compared using the *χ*^2^ test or Fisher’s exact test where appropriate. Statistical significance was defined as *p* < 0.05. All statistical analyses were performed in SPSS version 27.0.1, and figures were generated using GraphPad Prism 10.

## Result

A total of 102 patients with suspected autoimmune encephalitis were initially screened. Following diagnostic evaluation, 15 patients were excluded for the following reasons: presence of other autoimmune encephalitis–associated antibodies (*n* = 6), confirmed infectious encephalitis or metabolic/psychiatric disorders (*n* = 5), or incomplete clinical and laboratory data insufficient to establish a reliable diagnosis (*n* = 4). Ultimately, 87 patients who fulfilled the diagnostic criteria and tested positive for LGI1-IgG antibodies in serum and/or CSF were included in the final analysis. Among them, 55 patients were positive for LGI1 antibodies in serum only, 4 patients were positive in CSF only, and 28 patients were positive in both serum and CSF.

### Demographic characteristics

Among the 87 patients diagnosed with anti-LGI1 antibody–positive autoimmune encephalitis, 62 were male (71.3%) and 25 were female (28.7%), yielding a male-to-female ratio of 2.48:1. The mean age at disease onset was 58.32 ± 13.20 years (Kolmogorov–Smirnov test: *p* = 0.082 > 0.05), with an age range of 20 to 95 years. The median follow-up duration was 21 months (range, 6–36 months). Detailed clinical information is presented in Table 1.

**Table 1:** Clinical data from a subset of patients with LGI1 encephalitis.

Patient code	Gender	Age	First symptoms	Hyponatremia	HHCY	Abnormal electrocardiogram	Abnormal thyroid function	Cerebrospinal fluid pressure (mmH_2_O)	White blood cell count in cerebrospinal fluid(*10^6^/L)	Abnormal cerebrospinal fluid proteins	First-line treatment plan	mRs Score
1	Male	84	Transient loss of consciousness	+	−	+	+	/	/	/	MPT IVIG	4 → 2
2	Male	51	Memory impairment	−	−	+	/	235	3	+	MPT IVIG	1 → 0
3	Female	60	Paroxysmal right limb twitching	−	−	+	−	90	1	−	MPT IVIG	1 → 0
4	Female	55	Paroxysmal limb twitching	−	−	+	−	135	1	−	IVIG	1 → 0
5	Male	71	Paroxysmal limb twitching	+	−	+	−	/	/	/	MPT IVIG	4 → Death
6	Female	43	Limb fatigue	+	−	−	+	140	10	−	MPT IVIG	3 → 1
7	Male	46	Involuntary generalized muscle contractions	−	+	+	+	107	1	+	MPT	1 → 1
8	Female	62	Memory impairment	+	−	+	−	/	/	/	MPT	4 → 3
9	Male	73	Paroxysmal shaking of the right shoulder	+	+	+	−	50	4	+	MPT	1 → 1
10	Male	71	Paroxysmal angular twitching	+	+	+	−	170	1	−	MPT IVIG	2 → 2
11	Male	52	Episodic dizziness	−	+	−	/	150	4	+	MPT IVIG	2 → 1
12	Male	51	Paroxysmal limb twitching	+	−	+	−	190	7	−	MPT IVIG	1 → 1
13	Male	53	Memory impairment	−	−	+	/	160	1	+	MPT IVIG	1 → 1
14	Male	84	Paroxysmal limb twitching	−	−	+	+	120	5	+	MPT IVIG	3 → 2
15	Female	51	Memory impairment	−	−	+	−	260	1	+	MPT IVIG	4 → 1
16	Male	59	Paroxysmal limb twitching	+	−	+	−	100	5	−	MPT	4 → 1
17	Male	20	Transient loss of consciousness	−	−	−	/	/	/	/	MPT IVIG	4 → 1
18	Male	71	Mental and behavioral disturbances	−	−	+	/	180	1	−	MPT IVIG	3 → 0
19	Male	39	Marked cognitive impairment	+	+	+	+	110	1	−	MPT IVIG	3 → 0
20	Female	49	Paroxysmal limb twitching	+	−	+	−	150	6	−	IVIG	4 → 0
21	Male	73	Epileptic seizures	−	−	/	/	148	2	−	MPT IVIG	4 → 1
22	Female	70	Drowsiness	+	−	/	+	50	4	+	IVIG	4 → 3
23	Male	71	Paroxysmal limb twitching	+	−	+	/	155	5	−	MPT IVIG	4→
24	Female	67	Memory impairment	−	/	/	+	185	21	−	MPT IVIG	1 → 0
25	Female	43	Memory impairment	+	−	−	−	140	1	−	MPT	1 → 0
26	Male	64	Memory impairment	+	+	/	−	100	30	+	MPT IVIG	1 → 1
27	Female	43	Paroxysmal limb twitching	+	−	+	/	140	10	−	MPT IVIG	3 → 1
28	Male	79	Memory impairment	−	/	−	−	125	7	+	MPT IVIG	1 → 4
29	Male	52	Memory impairment	−	−	/	/	130	5	+	MPT	1 → 1
30	Male	72	Memory impairment	+	−	/	−	130	6	+	MPT	3 → 1
31	Male	49	Memory impairment	−	+	−	/	170	2	−	MPT IVIG	1 → 0
32	Female	59	Memory impairment	−	−	/	+	160	1	−	MPT	2 → 1
33	Female	40	Epileptic seizures	+	+	/	/	145	12	+	MPT IVIG	1 → 1
34	Male	76	Weakness in the limbs	+	−	/	/	180	12	+	MPT IVIG	4 → 2
35	Male	68	Memory impairment	+	+	−	−	150	1	+	MPT IVIG	2 → 2
36	Female	52	Psychomotor slowing	−	−	/	/	175	9	+	MPT IVIG	3 → 1
37	Female	34	Epileptic seizures	+	/	/	−	135	1	−	MPT IVIG	1 → 2
38	Male	62	Memory impairment	+	−	/	−	110	5	+	IVIG	1 → 1
39	Male	71	Memory impairment	+	−	/	/	165	3	−	MPT IVIG	5 → 1
40	Male	57	Epileptic seizures	+	−	/	/	125	5	+	MPT IVIG	3 → 1
41	Male	63	Memory impairment	−	+	+	/	120	2	−	MPT IVIG	1 → 2
42	Male	61	Epileptic seizures	+	−	/	−	145	1	−	MPT IVIG	1 → 1
43	Male	62	Mental and behavioral disturbances	+	/	/	/	160	4	−	MPT IVIG	1 → 1
44	Male	33	Involuntary movement	−	+	/	−	165	6	−	IVIG	1 → 0
45	Female	69	Epileptic seizures	+	−	−	/	160	3	−	IVIG	1 → 0
46	Male	43	Involuntary movement	−	+	+	−	130	18	+	IVIG	2 → 2
47	Male	62	Mental and behavioral disturbances	+	−	/	/	115	4	−	MPT IVIG	2 → 2
48	Male	66	Epileptic seizures	+	−	/	−	95	4	−	MPT IVIG	3 → 0
49	Male	52	Transient loss of consciousness	−	−	/	/	90	5	−	MPT	1 → 0
50	Female	95	Memory impairment	+	−	/	−	50	3	+	IVIG	4 → 4
51	Female	52	Psychomotor slowing	+	+	−	−	160	6	−	MPT IVIG	1 → 1
52	Male	33	Epileptic seizures	+	−	/	/	175	4	−	MPT IVIG	5 → 0
53	Male	45	Mental and behavioral disturbances	−	/	−	−	180	7	−	MPT IVIG	1 → 0
54	Female	59	Mental and behavioral disturbances	+	−	/	/	190	4	−	MPT IVIG	4 → 1
55	Male	61	Epileptic seizures	−	−	/	−	155	2	−	MPT IVIG	1 → 0
56	Female	44	Mental and behavioral disturbances	/	+	+	−	115	6	−	MPT IVIG	1 → 2
57	Male	65	Epileptic seizures	−	−	/	/	150	6	−	MPT IVIG	1 → 1
58	Male	43	Paroxysmal limb numbness	+	−	/	−	120	3	+	MPT IVIG	2 → 1
59	Female	70	Limb pain and numbness	−	−	/	−	125	17	−	MPT IVIG	1 → 0
60	Male	49	Epileptic seizures	−	+	/	/	200	5	+	MPT IVIG	1 → 1
61	Male	67	Epileptic seizures	+	−	/	/	130	6	−	MPT IVIG	2 → 1
62	Male	62	Epileptic seizures	/	−	−	−	125	2	−	MPT IVIG	3 → 2
63	Male	51	Memory impairment	+	−	/	−	145	7	+	MPT IVIG	1 → 1
64	Female	66	Epileptic seizures	+	+	/	−	165	6	−	MPT IVIG	3 → 2
65	Female	61	Mental and behavioral disturbances	−	−	/	−	150	2	−	MPT IVIG	2 → 1
66	Male	67	Autonomic dysfunction	+	/	−	/	120	4	−	MPT IVIG	1 → 1
67	Male	44	Epileptic seizures	−	−	/	/	165	1	−	MPT IVIG	1 → 0
68	Female	59	Memory impairment	+	+	+	+	145	1	−	/	2 → 1
69	Male	61	Memory impairment	+	−	−	/	120	4	−	MPT IVIG	2 → 1
70	Male	69	Epileptic seizures	−	−	/	−	160	7	−	MPT IVIG	2 → 1
71	Male	75	Advanced intelligence decline	−	+	/	−	170	22	+	MPT IVIG	3 → 1
72	Female	44	Mental and behavioral disturbances	−	−	/	/	50	13	−	MPT IVIG	2 → 1
73	Female	68	Memory impairment	+	−	−	−	115	5	−	IVIG	2 → 1
74	Female	46	Mental and behavioral disturbances	−	−	/	/	165	2	+	MPT IVIG	5 → 2
75	Male	50	Sleep dysfunction	/	−	/	/	170	4	−	IVIG	1 → 1
76	Female	38	Transient loss of consciousness	−	+	+	−	100	7	−	MPT IVIG	1 → 1
77	Male	71	Memory impairment	−	−	/	−	155	6	−	MPT IVIG	3 → 1
78	Male	55	Involuntary movement	−	/	/	−	95	2	−	MPT IVIG	1 → 1
79	Male	54	Involuntary movement	+	+	/	−	125	19	+	MPT IVIG	3 → 2
80	Female	51	Epileptic seizures	−	−	−	/	160	1	−	MPT IVIG	1 → 1
81	Male	65	Paroxysmal limb numbness	/	−	+	/	145	1	+	MPT IVIG	4 → 2
82	Male	52	Autonomic dysfunction	−	+	/	−	135	4	+	MPT IVIG	1 → 1
83	Male	65	Epileptic seizures	−	−	/	−	110	203	+	MPT IVIG	2 → 0
84	Male	69	Involuntary movement	−	−	/	−	170	6	+	IVIG	1 → 0
85	Female	63	Epileptic seizures	/	+	+	/	155	3	−	MPT IVIG	1 → 0
86	Male	63	Memory impairment	−	−	/	−	125	300	−	IVIG	3 → 1
87	Male	65	Memory impairment	−	+	−	−	85	2	−	MPT IVIG	5 → 0

“/”, No result seen; “−”, No; “+”, YES; mRS, Modified Rankin Scale.

The first score on the left side of the arrow is the score at admission, and the score on the right side of the arrow is the score at the last follow-up; IVIG, immunoglobulin; MPT, Methylprednisolone; HHCY, Hyperhomocysteinemia.

#### Clinical characteristics

As of the last follow-up on March 1, 2025, clinical data from 87 patients with anti-LGI1 encephalitis were analyzed. Memory impairment was observed in 69 patients (79.3%), and 60 patients (69.0%) experienced epileptic seizures, including 39 focal seizures and 21 tonic–clonic seizures. Mental and behavioral disturbances occurred in 51 patients (58.6%), sleep dysfunction in 39 (44.8%), involuntary movements in 37 (42.5%), and impaired consciousness in 25 (28.7%). Autonomic dysfunction was present in 24 patients (27.4%), including hyperhidrosis (*n* = 9), defecation difficulties (*n* = 4), urinary dysfunction (*n* = 4), cardiac arrhythmias (*n* = 3), central hypoventilation (*n* = 3), and diarrhea (*n* = 1). Speech impairment occurred in 22 patients (25.3%). FBDS were identified in 14 patients (16.1%). Other less common symptoms included limb numbness (*n* = 6, 6.9%), limb weakness (*n* = 4, 4.6%), tumors (*n* = 4, 4.6%; 1 gastric signet ring cell carcinoma, 1 pituitary adenoma, and 2 prostate cancers), visual disturbances (*n* = 3, 3.4%; 2 reduced visual acuity, 1 diplopia), ataxia (*n* = 2, 2.3%), and intestinal obstruction (*n* = 2, 2.3%). Rare manifestations included muscle fasciculations (*n* = 1, 1.1%), choking while drinking water (*n* = 1, 1.1%), and dysphagia (*n* = 1, 1.1%). A total of 18 patients (20.7%) experienced relapse, and 8 patients (9.2%) required admission to the intensive care unit (ICU).

### Laboratory and electrocardiographic findings

**Figure 1:**
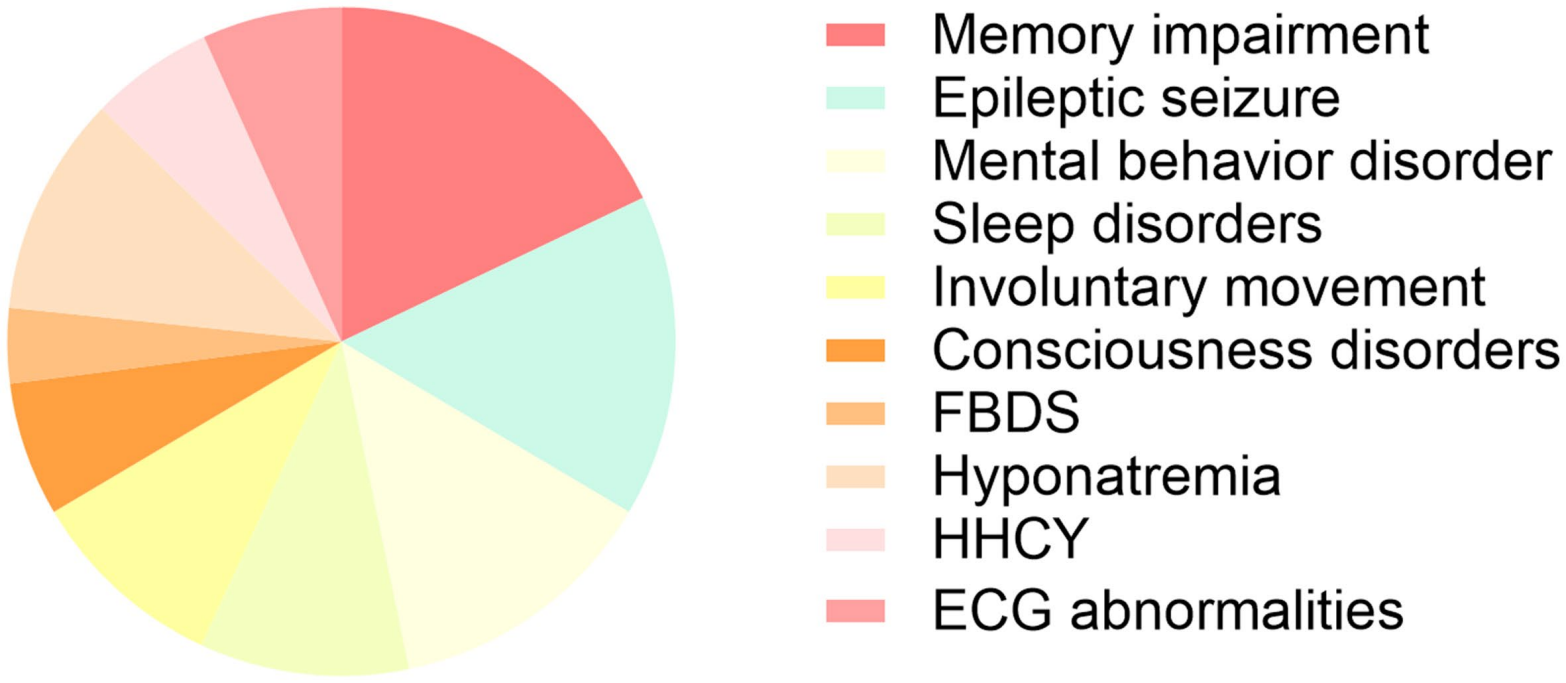
Pie chart illustrating the distribution of typical clinical symptoms by case number in patients with anti-LGI1 encephalitis.

Thyroid function tests were available in 53 patients, among whom 9 (17.0%) showed abnormalities. Carcinoembryonic antigen (CEA) levels were measured in 13 patients, with 3 (23.1%) exhibiting elevated values. Serum sodium levels were obtained from 82 patients, of whom 41 (50.0%) had hyponatremia, with a median sodium level of 129.00 mmol/L [interquartile range (IQR): 123.25–133.00 mmol/L]. Homocysteine levels were assessed in 80 patients, and 23 (28.7%) presented with HHCY. Lumbar puncture data during the acute phase were available in 68 patients. Elevated CSF opening pressure (>180 mmH₂O) was observed in 10 patients (14.7%), with a median pressure of 190 mmH₂O (IQR: 185–235; 1 mmH₂O = 9.8 Pa). CSF pleocytosis was present in 22 patients (32.4%), with a median white blood cell count of 18.5 × 10^6^/L (IQR: 12.75–73.25). Elevated CSF protein levels were noted in 27 patients (39.7%), with a median concentration of 0.621 g/L (IQR: 0.530–0.710). Increased CSF glucose levels were detected in 18 patients (26.5%), with a median of 5.565 mmol/L (IQR: 5.005–6.357). CSF chloride levels were decreased in 31 patients (45.6%), with a median of 114 mmol/L (IQR: 110–116). Oligoclonal bands (OCBs) were assessed in 39 patients, with 5 (12.8%) demonstrating CSF-specific OCBs. Electrocardiogram (ECG) data were available for 54 patients. After excluding individuals with pre-existing cardiovascular disease (8 with coronary artery disease, 1 with valvular heart disease, 1 with arrhythmia, and 2 post–coronary intervention), 42 patients were included in the final ECG analysis. ECG abnormalities were defined as ST-segment and/or T-wave abnormalities in one or more leads. A summary of key clinical and laboratory characteristics is presented in Table 2 and Figure 1.

**Table 2:** Summary of characteristic clinical manifestations and laboratory test results of 87 patients.

Clinical manifestation	Example (%)
Memory impairment	69 (79.3)
Epileptic seizures	60 (69.0)
Mental and behavioral disturbances	51 (58.6)
Hyponatremia	41 (50.0)
Sleep dysfunction	39 (44.8)
Involuntary movement	37 (42.5)
Impaired consciousness	25 (28.7)
Autonomic dysfunction	24 (27.4)
HHCY	23 (26.4)
Speech impairment	22 (25.3)
FBDS	14 (16.1)

FBDS, Faciobrachial dystonic seizure; HHCY, Hyperhomocysteinemia.

**Figure 2:**
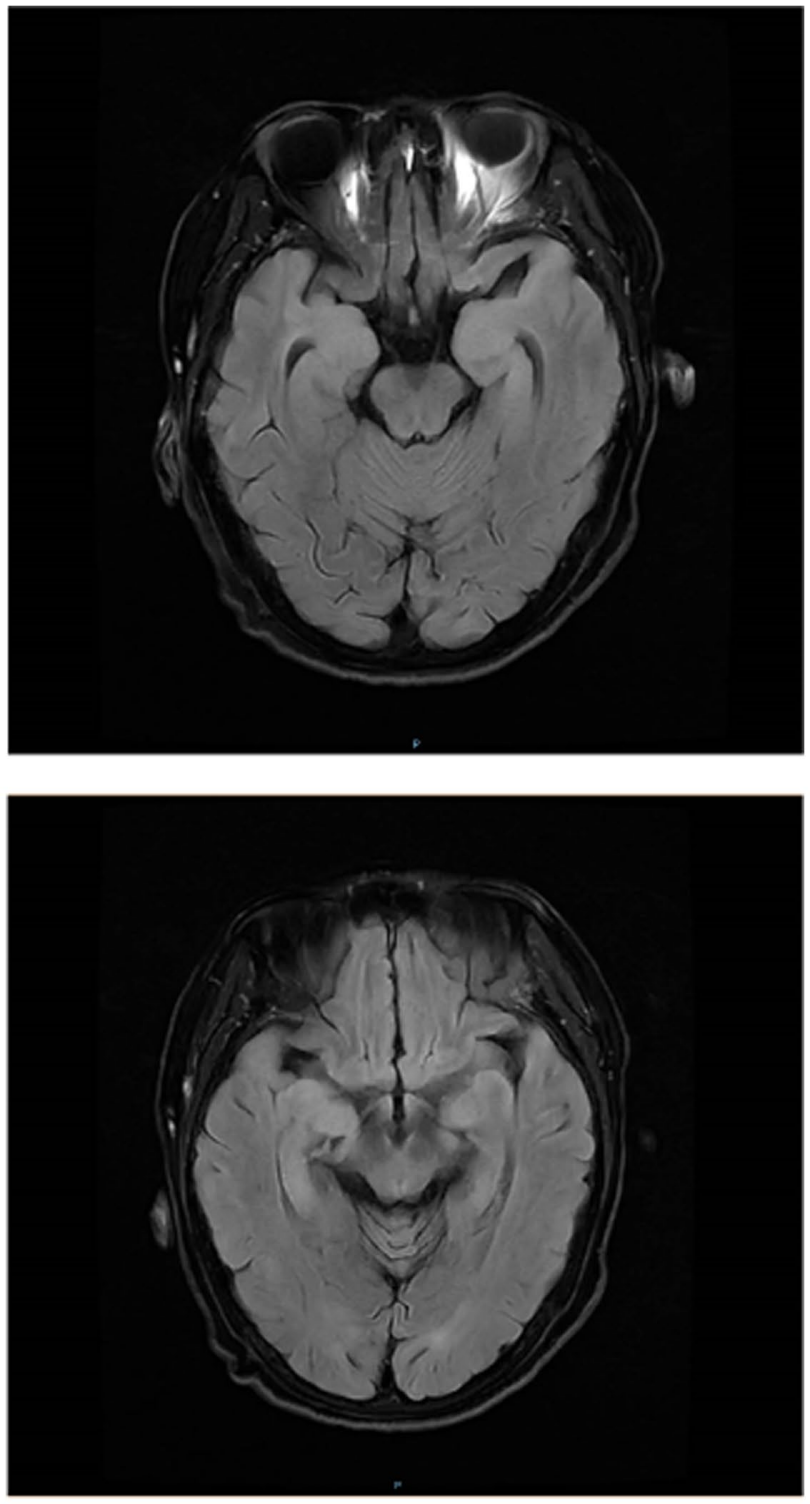
The above depict the head magnetic resonance imaging (MRI) findings of patients with confirmed anti-LGI1 encephalitis. These images demonstrate T2-FLAIR hyperintensities in the bilateral amygdala, hippocampus, and insular lobe.

**Figure 3:**
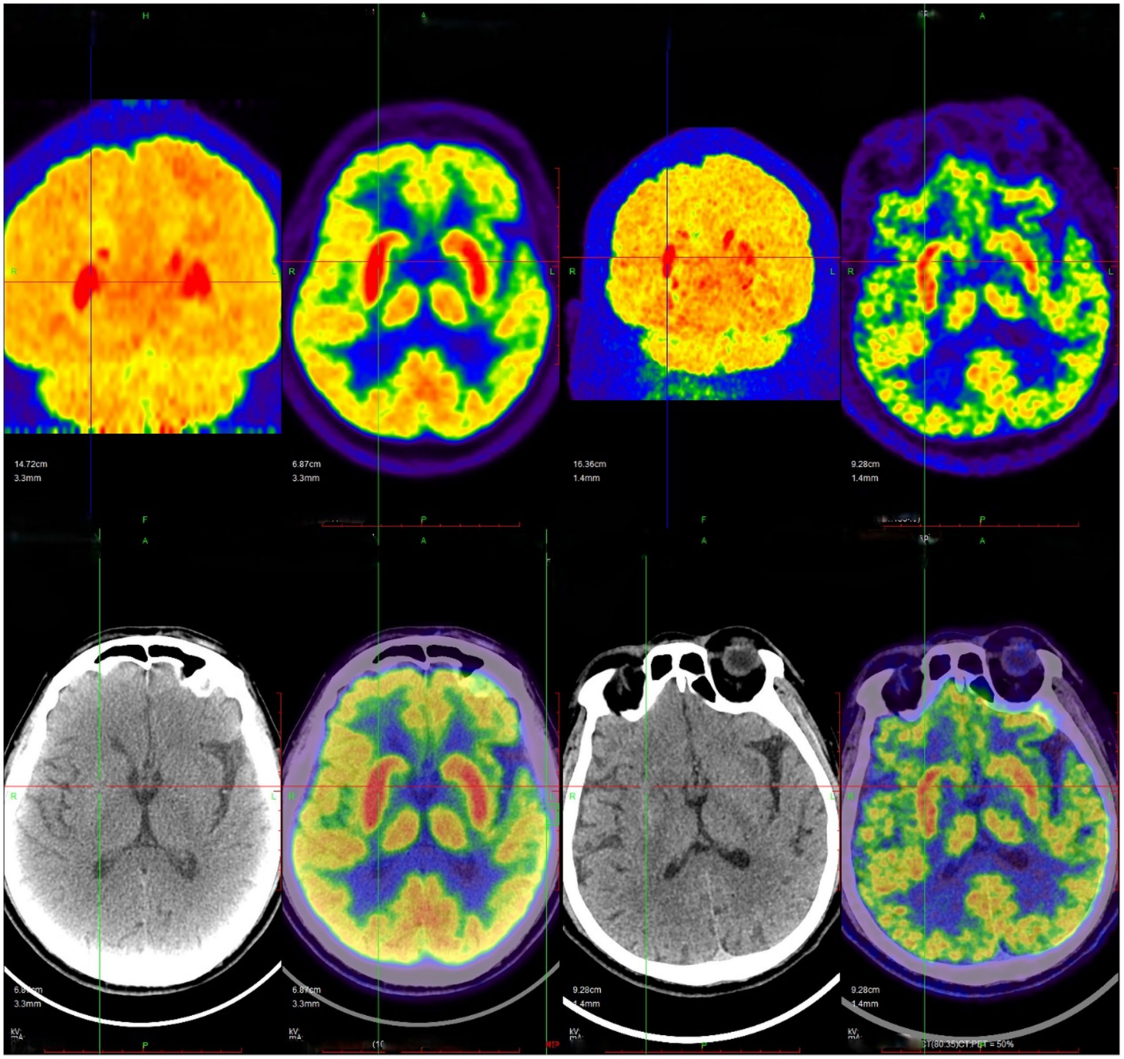
18F-FDG PET/CT images of the patient with anti-LGI1 encephalitis. The images demonstrate elevated metabolism in the bilateral basal ganglia, with a more pronounced increase on the right side. Additionally, there is a mildly increased metabolism in the bilateral hippocampi, with a relatively more notable elevation on the right side.

Brain MRI and 18F-FDG PET/CT Findings: Brain magnetic resonance imaging (MRI) was performed in 80 patients, of whom 75 (93.8%) demonstrated abnormal findings, predominantly characterized by T2 FLAIR hyperintensity involving the mesial temporal lobe (T2 FLAIR-MTL) (representative images are shown in Figure 2). Temporal lobe involvement was the most frequent imaging abnormality, observed in 44 patients (55.0%), including 15 (18.7%) with left-sided involvement, 8 (10.0%) with right-sided involvement, and 20 (25.0%) with bilateral involvement. Hippocampal abnormalities were identified in 41 patients (51.2%), of whom 8 (10.0%) had left-sided involvement, 7 (8.7%) had right-sided involvement, and 26 (32.5%) had bilateral involvement. Amygdalar involvement was present in 9 patients (11.2%), including 1 (1.2%) left-sided, 2 (2.5%) right-sided, and 6 (7.5%) bilateral cases. Insular cortex lesions were detected in 5 patients (6.2%), and frontal lobe involvement was noted in 7 patients (8.7%). A single patient (1.2%) exhibited abnormalities in the cingulate gyrus. Detailed MRI findings are summarized in Table 3. 18F-FDG PET/CT imaging was performed in 39 patients. Increased glucose metabolism was observed in the hippocampus in 21 patients (53.8%), the basal ganglia in 25 patients (64.1%), the temporal lobe in 8 patients (20.5%), and the striatum in 8 patients (20.5%). Additionally, hypermetabolism of the insular cortex was detected in 4 patients (10.3%), and increased uptake in the putamen or caudate nucleus was also found in 4 patients (10.3%). Representative PET/CT images demonstrating these metabolic patterns are shown in Figure 3.

**Table 3:** The cumulative sites of head magnetic resonance imaging(MRI)in 80 patients.

Part		Example (%)
Temporal lobe	Left	15 (18.7)
	Right side	8 (10.0)
	Bilateral	20 (25.0)
Seahorse	Left	8 (10.0)
	Right side	7 (8.7)
	Bilateral	26 (32.5)
Amygdala	Left	1 (1.2)
	Right side	2 (2.5)
	Bilateral	6 (7.5)
Insular cortex	Left	2 (2.5)
	Right side	1 (1.2)
	Bilateral	2 (2.5)
Cingulated gyrus	Left	1 (1.2)
	Right side	/
	Bilateral	/
Frontal lobe	Left	2 (2.5)
	Right side	2 (2.5)
	Bilateral	3 (3.7)

“/”: indicates non-accumulation.

### Treatment and follow-up

All patients received first-line immunotherapy during hospitalization. Among them, 55 patients received combination therapy with corticosteroids and intravenous immunoglobulin (IVIG) during the acute phase, 11 patients were treated with corticosteroids alone, and 11 patients received IVIG monotherapy. Notably, in the corticosteroid-only and IVIG-only groups, therapy was initiated at the early stage immediately after diagnosis. In the combination therapy group, treatment was typically initiated with one agent, and the second agent was added within 3–7 days if clinical improvement was insufficient. For 10 patients who showed inadequate clinical response after approximately 2 weeks of first-line therapy, second-line immunotherapy with cyclophosphamide was administered. Upon discharge, patients who had received corticosteroids (either alone or in combination with IVIG) continued oral corticosteroid tapering in the outpatient setting (with a reduction of one to two tablets per week), either alone or in combination with oral mycophenolate mofetil. Patients who had received IVIG monotherapy during the acute phase were prescribed oral mycophenolate mofetil for maintenance therapy after discharge (in the management of anti-LGI1 encephalitis, corticosteroids and IVIG are commonly employed as first-line therapies, which have been demonstrated to effectively alleviate acute symptoms and reduce relapse rates. In our cohort, some patients received second-line therapies, such as cyclophosphamide. Although immunosuppressants, including azathioprine, are frequently used in other immune-mediated disorders, there is currently insufficient evidence to support their efficacy in anti-LGI1 encephalitis; therefore, these agents were not administered in our study). By the last follow-up, a total of 18 patients had experienced relapse, including 13 in the corticosteroid + IVIG group, 3 in the corticosteroid-only group, and 2 in the IVIG-only group, corresponding to relapse rates of 23.6% (13/55), 27.3% (3/11), and 18.2% (2/11), respectively. Follow-up data were available for 63 patients. Among these, 3 patients (4.8%) exhibited a poor prognosis (mRS > 2), and 1 patient (1.6%) had died. The remaining 59 patients (93.7%) had a favorable prognosis (mRS ≤ 2).

## Discussion

LGI1 is a neuronally secreted protein involved in the regulation of synaptic function (6). Previous studies have demonstrated that intracerebroventricular injection of IgG antibodies from anti-LGI1-positive patients into mice significantly reduces the expression levels of hippocampal potassium channels and AMPA receptors (AMPARs), leading to increased neuronal excitability and impaired memory function (7). These findings provide strong evidence supporting the direct pathogenic role of LGI1 antibodies. It is noteworthy that anti-LGI1 encephalitis differs from other more common forms of AE, such as anti-NMDAR encephalitis and paraneoplastic encephalitis, in terms of clinical presentation, laboratory findings, and neuroimaging features. Based on a cohort of 87 patients diagnosed with anti-LGI1 encephalitis, the present study systematically analyzed their clinical characteristics to enhance understanding of this rare autoimmune neurological disorder.

Epidemiological data suggest that anti-LGI1 antibody-associated encephalitis predominantly affects middle-aged and elderly individuals. In our cohort, the age at disease onset ranged from 20 to 95 years, with a mean onset age of 58.32 ± 13.19 years. Among the 87 patients, 62 (71.3%) were male and 25 (28.7%) were female. This sex distribution aligns with previous studies, which have consistently reported a male predominance of approximately 60–70% among patients with anti-LGI1 encephalitis (8–10).

In terms of clinical manifestations memory impairment and seizures were the predominant initial manifestations in patients with anti-LGI1 encephalitis, while FBDS, involuntary movements, and autonomic dysfunction frequently appeared as accompanying or progressive symptoms. Memory impairment (69/87, 79.3%) was the most common clinical feature and often constituted the first noticeable symptom. This may be related to the pathogenic effects of LGI1 antibodies on neuronal excitability and synaptic plasticity, the latter playing a critical role in learning and memory processing. Seizures (60/87, 69.0%) represented the second most prevalent clinical manifestation. In most cases, the seizures were characterized by focal onset, typically originating from medial temporal lobe structures such as the hippocampus and amygdala (11).

Interestingly, FBDS, a hallmark clinical feature of anti-LGI1 encephalitis, was identified in only 16.1% (14/87) of patients in our cohort, which is notably lower than the 30–50% reported in previous studies (8–10, 12). This discrepancy may be attributed to the characteristic features of FBDS: episodes are extremely brief (typically lasting only a few seconds), occur with high frequency (up to 50–80 episodes per day), and primarily involve the face and upper limbs, occasionally extending to the lower limbs. These features can lead to underrecognition in clinical settings (13). The nature of FBDS remains debated, with uncertainty as to whether it represents a seizure subtype, an independent phenomenon, or a form of dystonia. Chen et al. (14) reported that some FBDS episodes were immediately followed by typical seizures—termed FBDS+—suggesting that FBDS+ may constitute a seizure variant. Clinically, FBDS must be distinguished from dystonia and conventional seizures. For suspected cases, early testing for autoimmune encephalitis-related antibodies is recommended.

In this study, 28.7% (23/80) of patients with anti-LGI1 encephalitis were found to have HHCY. Follow-up evaluations showed that these patients generally exhibited favorable clinical outcomes, with a median mRS score of 1. Additionally, comparative analyses between patients with and without HHCY revealed no statistically significant differences in comorbidities, clinical prognosis, or relapse rates (*p* > 0.05). Previous studies have demonstrated that HHCY is associated with multiple neurodegenerative disorders, including Alzheimer’s disease, Parkinson’s disease, Lewy body dementia, and multiple sclerosis, and has been recognized as an independent risk factor for cognitive decline (15). Moreover, plasma homocysteine levels have been reported to be negatively correlated with cognitive performance. However, the relationship between HHCY and anti-LGI1 encephalitis remains insufficiently understood. Further studies are needed to clarify the potential mechanistic involvement and clinical relevance of HHCY in the pathogenesis and prognosis of this disease.

In terms of laboratory findings, hyponatremia was observed in 50% (41/82) of patients in this study, which is consistent with previously reported incidence rates (16, 17). The underlying pathophysiological mechanism may be associated with syndrome of inappropriate antidiuretic hormone secretion (SIADH), potentially secondary to hypothalamic involvement caused by central nervous system inflammation (17, 18). Notably, thyroid dysfunction was identified in 17.0% (9/53) of patients, which is slightly lower than rates reported in earlier studies (19). This discrepancy may be attributed to the relatively small sample size in our cohort. Previous research has shown that Hashimoto’s encephalopathy (HE) can also present with psychiatric symptoms, memory impairment, seizures, and cognitive dysfunction, and typically responds favorably to corticosteroid therapy (20). However, HE is generally characterized by elevated antithyroid antibodies, and thus must be carefully differentiated from autoimmune encephalitis in clinical practice.

Regarding electrocardiographic (ECG) findings, after excluding patients with pre-existing cardiac disease or hypertension, 42 patients remained for analysis. Among them, 26 patients (26/42, 61.9%) demonstrated ECG abnormalities. Specifically, 35.7% (15/42) exhibited ST-segment abnormalities in certain leads at admission, and 26.2% (11/42) showed T-wave abnormalities. These changes may be associated with LGI1 antibody–mediated dysfunction of potassium channels or may reflect seizures-related autonomic dysregulation leading to transient arrhythmias. Previous studies have reported that autoimmune encephalitis may be associated with sinoatrial node dysfunction (21). In our study, although 26 patients exhibited ECG abnormalities, none presented with corresponding cardiac symptoms. This discrepancy may be related to differences in patient characteristics or regional variability. Dürr et al. (22) reported a case of LGI1 antibody–positive encephalitis resulting in myocardial ischemia and sudden cardiac death despite angiographically normal coronary arteries. Therefore, even in the absence of structural heart disease, patients with LGI1 antibody positivity should undergo prompt ECG assessment upon admission to monitor for potential cardiac complications.

In terms of CSF characteristics, anti-LGI1 antibody–associated encephalitis typically shows only mild inflammatory changes. In this study, 14.7% (10/68) of patients demonstrated elevated CSF opening pressure (>180 mmH₂O), 32.4% (22/68) exhibited CSF pleocytosis (>8 × 10^6^/L), and 39.7% (27/68) had increased CSF protein concentrations (>0.45 g/L). Additionally, abnormalities in CSF glucose and chloride levels were observed in a subset of patients. The CSF profile in anti-LGI1 encephalitis may resemble that of certain viral encephalitides; therefore, careful differentiation from infectious etiologies is required in clinical practice. Compared with anti-NMDAR encephalitis, patients with anti-LGI1 encephalitis generally present with lower CSF white blood cell counts and milder blood–brain barrier disruption (23), which may reflect a more targeted immune response against neuronal surface LGI1 antigens rather than diffuse neuroinflammatory infiltration.

Regarding imaging features, 93.8% (75/80) of patients demonstrated T2 FLAIR-MTL in the medial temporal lobe, with predominant involvement of the temporal lobe (44/75, 58.7%) and hippocampus (41/75, 54.7%), consistent with the findings reported by Cornacchini et al. (24). Notably, five patients (5/80, 6.2%) in the acute phase exhibited normal MRI findings, which may be attributed to early and timely initiation of immunotherapy. This observation suggests that a normal MRI does not exclude the diagnosis of anti-LGI1 encephalitis, and diagnostic evaluation should incorporate clinical presentation, autoimmune encephalitis antibody testing, and cerebrospinal fluid analysis. With respect to 18F-FDG PET/CT, previous studies and case reports have demonstrated that anti-LGI1 encephalitis frequently presents with hypermetabolism in the basal ganglia and/or medial temporal lobes (25, 26), a pattern consistent with the results of this study. Liang et al. (26), further reported that PET imaging has higher sensitivity than MRI in detecting early metabolic abnormalities. Additionally, 18F-FDG PET/CT enables simultaneous screening for underlying neoplasms, given that a subset of autoimmune encephalitis cases may be paraneoplastic. Therefore, whole-body 18F-FDG PET/CT is recommended as part of the diagnostic workup.

In our study, combination therapy with corticosteroids and IVIG resulted in more rapid clinical improvement compared with corticosteroid monotherapy or IVIG alone. This finding is consistent with previous reports by Teng et al. (27) and Rodriguez et al. (9), which demonstrated greater efficacy of combination therapy over single-agent treatment. In our study, seven patients experienced relapse after discontinuation of corticosteroids following symptomatic improvement (the duration of corticosteroid therapy in these patients was 3, 4, 6, 7, 10, 12, and 14 months, respectively), highlighting the ongoing uncertainty regarding the optimal duration of immunotherapy, Large multicenter studies are needed to clarify treatment withdrawal strategies and relapse-related factors. In this study, the median mRS score at admission was 2 (IQR 1–3), which improved to 1 (IQR 0–1) at the final follow-up. Statistical analysis confirmed significant functional improvement after immunotherapy (*p* < 0.001), indicating a generally favorable prognosis in anti-LGI1 encephalitis. Although most patients (93.7%, 59/63) achieved good outcomes with timely treatment, previous studies have reported deaths among untreated patients (28), highlighting the increased mortality risk associated with delayed diagnosis and therapy. Therefore, early recognition and standardized immunotherapy are essential not only for controlling acute symptoms but also for improving long-term outcomes. Establishing rapid diagnostic and treatment pathways in clinical settings is crucial to maximizing therapeutic benefit within the optimal treatment window.

We conducted a comparative analysis between the relapse and non-relapse groups to identify potential factors associated with disease recurrence. The variables included in the analysis were sex, age, clinical manifestations, the presence of HHCY, hyponatremia, electrocardiographic abnormalities, thyroid dysfunction, neuroimaging lesion distribution, and treatment regimen. However, no statistically significant differences were observed between the two groups (*p* > 0.05), which may be due to the relatively small number of relapse cases in our cohort. Therefore, future studies with larger sample sizes are needed to further investigate and validate potential risk factors for relapse.

Previous studies on anti-LGI1 encephalitis have primarily reported that its main clinical manifestations include memory impairment, seizures, psychiatric and behavioral abnormalities, FBDS, and hyponatremia. In the present study, we further observed that a subset of patients in the acute phase developed electrocardiographic abnormalities and HHCY. Although previous case reports or small-sample studies have suggested that patients with anti-LGI1 encephalitis may experience cardiac or metabolic complications (19), the specific prevalence rates of HHCY and electrocardiographic abnormalities have not been widely quantified. However, our study confirms and quantifies these associations, providing valuable data to complement the existing body of research. Moreover, our cohort included patients from two tertiary hospitals, resulting in a relatively large sample size that is rarely achieved in prior studies. To ensure diagnostic accuracy and analytical robustness, patients with incomplete medical records or coexisting antibodies were excluded. Nonetheless, such strict inclusion criteria may have introduced selection bias. This limitation, combined with the retrospective study design, may restrict the generalizability of our findings. Therefore, future prospective, multicenter studies with broader inclusion criteria are warranted to validate these results.

This study has several limitations. First, as a retrospective study conducted primarily at tertiary medical centers, potential selection and referral biases may exist. Second, because most patients declined repeat serum and cerebrospinal fluid antibody testing during follow-up for economic or other reasons, it was not feasible to evaluate the relationship between dynamic changes in antibody titers and clinical outcomes. Third, the sample size for certain subgroup analyses was relatively small, which may limit the generalizability of our findings. Finally, this study did not specifically analyze the clinical characteristics of relapsed patients, which will be an important focus of future prospective investigations.

## Data Availability

The original contributions presented in the study are included in the article/supplementary material, further inquiries can be directed to the corresponding authors.
